# Survey on knowledge of non-alcoholic fatty liver disease (NAFLD) among doctors in Sri Lanka: a multicenter study

**DOI:** 10.1186/s13104-018-3673-2

**Published:** 2018-08-03

**Authors:** Anne Thushara Matthias, Anthony Nilesh Ranjeev Fernandopulle, Suranjith L. Seneviratne

**Affiliations:** 1 National Hospital of Sri Lanka, No 33, Police Park Avenue, Colombo 05, Sri Lanka; 2 Teaching Hospital Jaffna, Jaffna, Sri Lanka; 3 Institute of Immunity and Transplantation, Royal Free Hospital and University College London, London, UK; 4 Department of Surgery, Faculty of Medicine, University of Colombo, Colombo, Sri Lanka

**Keywords:** Fatty liver, Liver steatosis, Steatohepatitis, NAFLD, Liver, Sri Lanka

## Abstract

**Objectives:**

There has been a global increase in the incidence and prevalence of NAFLD. We assessed the knowledge and awareness of NAFLD among gastroenterology doctors in three state sector hospitals.

**Results:**

80 medical officers and 58 post-graduate trainee doctors/consultants responded. 110 (79.7%) considered NAFLD a major health problem whilst 97 (70.3%) thought the prevalence of NAFLD was 10–40%. 52.9% saw 12–24 patients with NAFLD/year. A vast majority knew the risk factors for NAFLD: 127 (92.7%) diabetes mellitus, 135 (97.8%) Obesity, 132 (95.7%) Dyslipidemia and 87 (63%) PCOS. The methods for diagnosis were recognized by: USS 132 (95.7%), MRI 34 (24.6%), transient elastography 23 (16.7%) and liver biopsy 88 (63.8%) while, 53 (38.4%) recognized the non-invasive methods available for diagnosis. The trends in referral were lower than expected: 85 (61.6%) refer to a Gastroenterologist/Physician, 53 (38.4%) to a Gym, 67 (48.6%) to a weight loss clinic and 45 (32.6%) to a dietician. Significantly more postgraduate trainee doctors: recognized the availability of non-invasive investigations for NAFLD (P = 0.01) and read guidelines on NAFLD (P = 0.02) compared to non-trainee doctors. As a whole, a majority (57.2%) had not attended a lecture or read a guideline on NAFLD. The barriers for management included: lack of confidence 70 (50.7%) and time constraints 58 (42%).

**Electronic supplementary material:**

The online version of this article (10.1186/s13104-018-3673-2) contains supplementary material, which is available to authorized users.

## Introduction

In recent years, non-alcoholic fatty liver disease (NAFLD) has been gaining wide spread interest within the scientific community. NAFLD is considered as one of the commonest causes of cirrhosis worldwide and is the commonest cause of liver disease in the west [1].

The prevalence of NAFLD in Sri Lanka is 32.6%. This rate was derived from a study done within an urban population (as part of the Ragama Health Study). It was undertaken in the Western province of Sri Lanka which is a highly urbanized part of the country [2]. The approximate prevalence rates in rural Sri Lanka are significantly lower at 18% [3].

Despite the enormous amount of studies on NAFLD, treatment options are still limited. Life style modifications including exercise [4] and diet have proven their benefit beyond doubt in reducing liver fibrosis. NAFLD is an emerging epidemic in Asia. Prevention seems to be the cornerstone in curbing NAFLD. Public health measures to reduce obesity, combat insulin resistance are at present the most promising treatment modalities for NAFLD.

Most of the time, patients with NAFLD are seen by non-gastroenterologists/hepatologists. Studies done elsewhere in the world have shown a low level of knowledge and awareness of NAFLD among doctors. Therefore, many cases of NAFLD may pass undiagnosed and thus the point at which life style modifications and correction of risk factors could be done, be missed.

As prevention is one of the principal management strategies for progression of NAFLD, adequate knowledge about the disease and its diagnosis are essential for doctors. In the literature, there is very little information on knowledge of doctors regarding NAFLD [5]. Assessment of knowledge helps in formulating appropriate policies for implementing educational programs, which should will help with enhancing knowledge, as previously shown for other chronic liver diseases [6].

## Main text

### Methods

A comprehensive anonymous questionnaire (Additional file 1) was given to specialists and specialist doctors in training at three major tertiary care hospitals in the country. The first part of the questionnaire assessed basic demographic data. The second part consisted of questions assessing knowledge of risk factors, complications, methods of diagnosis, management options, progression and screening of NAFLD. The questionnaire content was tested by doing a pilot study on 25 medical officers. Some responses were open ended and others multiple choice in format, which were coded according to predetermined criteria. The study was approved by the Ethics review Committee of the National Hospital of Sri Lanka and the Post Graduate Institute of Medicine.

Data analysis was done using SPSS (version 17), continuous variables were shown as means and median and categorical variables were assessed using Chi square.

### Results

#### Demographic data of the respondents

The study consisted of 80 grade medical officers and junior doctors and 58 Post Graduate trainee doctors and consultants. The response rate was 100%. There were 62 females (44.9%) and 75 males (54.3%). A majority of participants 52.9% had consultations with 12–24 NAFLD patients per year and another 21% saw 24–36 patients per year.

#### Awareness about prevalence and gravity of the problem

A vast majority 110 (79.7%) considered NAFLD to be a major health problem. 97 (70.3%) thought NAFLD prevalence was between 10 and 40%. Of them, 50% thought it was between 10 and 30% and 20.3% thought it was between 30 and 40%.

#### Awareness of the factors associated with NAFLD

As for the indications for screening for NAFLD, there was very high knowledge in identifying the traditional associations: 127 (92.7%) for DM, 90 (65.2%) for hypertension, 135 (97.8%) for obesity, 121 (87.7%) for high AST and ALT, 132 (95.7%) for dyslipidemia and 87 (63%) for Polycystic Ovarian Syndrome (PCOS). There was moderate knowledge in identifying emerging associations: obstructive sleep apnoea 60 (43.5%) and hypothyroidism 80 (58%). 56 (40.6%) thought alcohol consumption was associated with NAFLD. The safe alcohol intake for male and females were thought to be less than 14 units for males by 71 (51.4%) and 7 units for females by 134 (97.1%).

#### Awareness about screening and diagnosis

Family screening for NAFLD was thought to be appropriate by 60 (43.5%) and 66 (47.8%) did not recommend it.

Table 1 shows the number of participants who identified the methods available for diagnosis of NAFLD.

**Table 1:** Methods available for diagnosis

Method of diagnosis	Number (percentage)
Ultrasound	132 (95.7%)
MRI	34 (24.6%)
TE	23 (16.7%)
Liver biopsy	88 (63.8%)

A smaller number 53 (38.4%) recognized that non-invasive methods were available for diagnosis of NAFLD. A minority of respondents 49 (35.1%) thought NAFLD could be diagnosed by elevated liver enzymes.

The correct indications for liver biopsy were identified by > 50% of the participants: 71 (51.4%) in patients at risk for steatohepatitis and cirrhosis, 70 (50.7%) if other conditions causing steatohepatitis cannot be excluded, 47 (34.1%) if other conditions causing steatohepatitis coexist, 26 (18.8%) if metabolic syndrome was present, 66 (47.8%) if NAFLD fibrosis score is high and 40 (29%) in children before starting pharmacological therapy.

#### Perception about complications and factors predicting severity

The factors that affect the severity of NAFLD were recognized by the participants as: older age 48 (34.8%), higher BMI 107 (77.5%), C Reactive Protein (CRP) 45 (32.6%), High INR 35 (25.4%), insulin resistance 102 (73.9%) and albumin 54 (34.9%).

NAFLD was not considered an inherited disorder by 61 (44.2%). 131 (94.9%) had the knowledge that NAFLD could lead to cirrhosis and the percentage that leads to cirrhosis as 10–30% was recognized by 72 (52.2%).

The drugs causing steatosis in the liver were reasonably recognized by the participants: Statins 56 (40.6%), valproate 76 (55.1%) paracetamol 69 (55%), amiodarone 57 (41.3%), methotrexate 82 (59.4%), steroids 47 (34.1%), Tamoxifen 42 (30.4) and Non steroidal anti inflammatory drugs (NSAIDs) 37 (26.8%). Two drugs which do not cause liver steatosis were identified by participants as culprits: azathioprine 40 (29%) and quinine 22 (15.9).

#### Trends in referrals

85 (61.6%) would refer patients to a gastroenterologist or to a physician. 53 (38.4%) would refer patients to a gym, 67 (48.6%) would refer to a weight loss clinic and 45 (32.6%) would refer to a dietician.

#### Management options

As for management options for NAFLD, most agreed on dietary modifications 130 (94.2%) and that exercise was crucial 123 (89.1%). A hypocaloric diet 24 (17.4%) was not recommended by many but most thought a low lipid diet 62 (44.9%) would help with management.

Management with Ursodeoxycholic acid, antioxidants, Obeticholic acid were identified by some as effective, though not recommended by guidelines. Avoiding alcohol consumption over the recommended limit was recognized by half the respondents [women 58 (42%) and men 60 (43.5%)]. Weight loss as an effective strategy was not recognized by many.

82 (59.4%) thought treatment of NAFLD should be initiated if high AST/ALT was noted, and 30 (21.7%) thought this should be after a liver biopsy.

A minority of participants 23 (16.7%) used Vitamin E for patients with NAFLD. In patients with established NAFLD, a majority were not reluctant to use statins 64 (46.4%).

#### Knowledge on NAFLD and barriers for management

As for engagement in continuous medical education on NAFLD, 79 (57.2%) did not attended a lecture or read a guideline. A minority 34 (24.6%) attended a lecture and read a guideline on NAFLD 21 (15.2%). The barriers for effective management of NAFLD as identified by the respondents included: lack of confidence 70 (50.7%), time constraints 58 (42%), extra cost 65 (47.1%), lack of compliance 85 (61.6%) and not been comfortable due to lack of knowledge of the subject 29 (21%).

#### Comparison of grade medical officer and junior doctors vs post graduate trainee doctors and consultants

There was no difference between the two groups in a majority of the knowledge aspects tested. The postgraduate trainee doctors recognized the availability of non-invasive investigations when compared with non-trainee doctors (P = 0.01) and read guidelines on NAFLD (P = 0.02).

### Discussion

Nonalcoholic fatty liver disease is a significant health problem and the number of patients having this condition is gradually increasing worldwide. The consequences of NAFLD include NASH, liver cirrhosis and hepatocellular carcinoma. This makes the condition a public health problem which needs urgent attention. This study explores the awareness and knowledge of NAFLD among doctors in a developing Asian country.

At present, definitive treatment modalities for NAFLD are not available, thus lifestyle changes including an increase in physical activity and a change in dietary habits are crucial. It is disappointing that only 32.6% of the respondents referred their NAFLD patients to a dietician. This low level of referrals has been seen in another study done in Poland where the referral rate was 30% [4]. With the establishment of an MD examination in Nutrition and placement of Dietitians in all major hospitals in the country, ignoring their valuable input into the management of this condition is indeed a pity. NAFLD is a metabolic disorder which needs lifestyle modifications. In order for patients to change their life style (especially diet), they would need the support that could be given by dieticians. Fat intake leads to hepatic fat deposition in animals but human data is not conclusive [5]. Most of the respondents thought low fat diet was beneficial as opposed to a low calorie diet. This points to doctors needing to acquire further knowledge on diet.

NAFLD is a distinct entity which needs specialist input as prevention of progression is the key. The low rate of referral to specialists seen in our study was also seen in another study done among GPs [5]. Seeking specialists input at an early stage could be beneficial for patients such as the identification of those needing specific therapy or a liver biopsy.

Our respondents had a fair knowledge on the merits of exercise and weight loss. According to the American guidelines [5] at least 3–5% of weight loss and if possible up to 10% is indicated and this seems to be the understanding among most of the participants of this study (65.2%). Weight loss reduces hepatic steatosis. Though the participants recognized weight loss to be crucial, follow up action such as referral to a gym, dietician or weight loss clinic was low.

At present Vitamin E as a therapy for NASH is recommended to patient with NASH proven by biopsy and who are non diabetic. This was known to our respondents but a majority would not use vitamin E. This may be due to the presumed ‘side effects’ in relation to of prostatic cancer and coronary artery disease. Strategies that are not recommended such as Metformin, Ursodeoxycholic acid (UDCA) were identified by a minority as effective. Half the respondents in our study believed that statins should be used for management of patients with NAFLD. Though statins are not contraindicated in NAFLD patients if they are indicated for another reason, statins as a management option for NAFLD is not recommended [5].

A synthetic farnesoid X receptor agonist, obeticholic acid, improved IR in the phase IIb FLINT trial. Issues related to safety and tolerability was raised, as there was an increase in LDL and significant pruritus. Therefore, according to the most recent European guidelines, they are not recommended for use in NASH [5]. A minority of the respondents believed family screening is recommended. The American guidelines do not recommend family screening [5].

Non-invasive methods for diagnosis are increasingly used for diagnosis of NAFLD. Knowledge on the availability of non-invasive methods to aid diagnosis was poor among the respondents. Continuous medical education on emerging modalities of diagnosis needs to be addressed.

The identification of common drugs causing hepatic steatosis was reasonable among the doctors. This contrasts with studies done elsewhere [6].

On the positive side, an overwhelming majority of respondents identified the association between the metabolic syndrome and NAFLD. This would lead to more screening for NAFLD and thus life style modifications. Screening of patients with metabolic risk factors for NAFLD is recommended by all major gastroenterology/hepatology associations and guidelines [5].

The principal barriers for NAFLD management as identified by the doctors included lack of knowledge (and thus confidence) on the subject and time constraints in management. This problem seems to be universal as doctors in the USA also reported similar constraints [6].

In conclusion this study advocates that better understanding of NAFLD is required and the best way forward would be continuous medical education of doctors on the subject.

## Limitations

The number of participants could have been larger to obtain a more representative view of all the doctors in Sri Lanka.

## Additional file

**Additional file 1.** Questionnaire. This was the questionnaire used for the research.

## References

[1] Bedogni G, Miglioli L, Masutti F, Tiribelli C, Marchesini G, Bellentani S (2005). Prevalence of and risk factors for nonalcoholic fatty liver disease: the Dionysos nutrition and liver study. Hepatology.

[2] Dassanayake AS, Kasturiratne A, Rajindrajith S, Kalubowila U, Chakrawarthi S, De Silva AP (2009). Prevalence and risk factors for non-alcoholic fatty liver disease among adults in an urban Sri Lankan population. J Gastroenterol Hepatol.

[3] Pinidiyapathirage MJ, Dassanayake AS, Rajindrajith S, Kalubowila U, Kato N, Wickremasinghe AR (2011). Non-alcoholic fatty liver disease in a rural, physically active, low income population in Sri Lanka. BMC Res Notes..

[4] Kargulewicz A, Stankowiak-Kulpa H, Grzymisławski M (2012). Assessment of the prevalence of nonalcoholic fatty liver disease among obese polish people and the estimation of the knowledge of Nutritional recommendations. Nowiny Lekarskie..

[5] Grattagliano I, D’Ambrosio G, D’Ambrozio G, Palmieri VO, Moschetta A, Palasciano G (2008). Improving nonalcoholic fatty liver disease management by general practitioners: a critical evaluation and impact of an educational training program. J Gastrointestin Liver Dis..

[6] Said A, Gagovic V, Malecki K, Givens ML, Nieto FJ (2013). Primary care practitioners survey of non-alcoholic fatty liver disease. Ann Hepatol..

