# Supplementary material for: Survey on knowledge of non-alcoholic fatty liver disease (NAFLD) among doctors in Sri Lanka: a multicenter study
Source: BMC Res Notes. 2018 Aug 3;11:556. doi: 10.1186/s13104-018-3673-2 (PMC6076419; doi:10.1186/s13104-018-3673-2)
Supplement: Supplementary file 1 — Additional file 1. Questionnaire. This was the questionnaire used for the research. [file 13104_2018_3673_MOESM1_ESM.pdf]

Questionnaire on knowledge of NAFLD among doctors

Desgnation(Circle)
- HO/RHO ☐
- MO/SHO ☐
- Consultant ☐
- Registrar ☐
- Senior Registrar ☐

Speciality (Circle one) Medicine/Gynaecology/Surgery

Subspecialty (Circle one) ☐

Qualification (Circle one) Diploma/Screening exam/Part 1/MD/MS/Mphil/PhD

Age
- <26 ☐
- 27-30 ☐
- 31-35 ☐
- 36-40 ☐
- 41-45 ☐
- >45 ☐

Gender: Male ☐ Female ☐

Years since passing out of faculty
- <5years ☐
- 5-10 years ☐
- 11-15 years ☐
- 15-20years ☐

Epidemiology/Scrreening/Diagnosis

Prevalence of NAFLD in Sri Lanka ☐ %

How many patietns with NAFLD do you see for a year? ☐

Who would you screen for NAFLD: (Tick all appropriate)

- ☐ DM
- ☐ HPT
- ☐ Obese
- ☐ Increased AST/ALT
- ☐ Dyslipidemia
- ☐ Sleep apnoea
- ☐ Hypothyroidism
- ☐ PCOS
- ☐ IHD
- ☐ Hypothyroidism

Do you screen patients with obesity or Diabetes for NAFLD? Yes ☐ No ☐

Is family screening for NAFLD recommeneded Yes ☐ No ☐

Methods available for diagnosis
- ☐ USS
- ☐ Thromboelastometry
- ☐ CT
- ☐ MRI
- ☐ Liver biopsy
- AST
- ALT

Indications for liver biopsy?
- ☐ At risk for steatohepatitis and cirrhosis
- ☐ Other condiitons causing steatohepaittis can't be excluded
- ☐ Other condiitons causing steatohepaittis coexist
- ☐ Presence of metabolic syndrome
- ☐ High NAFLD fibrosis score
- ☐ In children before starting pharmacological therapy

Do you refer patients with NAFLD to a gastroenterologist/physician? Yes/No ☐

Managemnt of NAFLD involves

- ☐ Dietary modifications
- ☐ Exercise
- ☐ Vitmain E for non diabetic patients
- ☐ Statins
- ☐ Antioxidants
- ☐ Obetocholic acid

	Metformin		Weight loss of3-5%
	Pioglitazones		Weight loss of 10%
	Avoiding hepatotoxic drugs		Weight loss of 2%
	Ursodeoxycholic acid (UDCA)		Bariatric patients without cirrhosis
	Bariatric surgery in cirrhosis		Avoididng> 14units of alcohol/week in men
	Avoiding >7Us of alcohol/wk in women		

Which of the following sentences about NAFLD is true?

It is not inherited	TRUE	FALSE	
It can lead to cirrhosis	TRUE	FALSE	what perecntage

Which is the best diet in a patient with NAFLD?

a. Hypocaloric diet

b. Low lipid content diet

c. Low carbohydrate content diet

d. High protein content diet

Have you read a guideline or visited a lectute on management of NAFLD

	No
	Read Guideline
	Attended Lecture
	Other

Which patients with NAFLD should receive pharmacological treatment

	High AST ALT
	After Liver biopsy

What are the dangerous side effects of vitamin E

	Know	If so specify	
	Don’t know		

Do you frequently use Viamin E for patients with NAFLD?	Yes	No
Can you give statins for patients with high AST/ALT?	Yes	No

Which of the following drugs can precipitate or worsen a NAFLD

	Statins		Amidarone		Azathiprine
	Valproate		MTX		NSAIDs
	Paracetamol		Steroids		Quinine
			Tamoxifen		

Do you think NAFLD is a major health problem Yes/No

<u>Barriers for NAFLD management ?</u>

Lack of confidecne in manging it	
Time constraints	
Cost of evaluation and treatment	
Lack of compliance by patient	
Not confortable to discuss obesity with patient	
